# Clinical trial recruitment in primary care: exploratory factor analysis of a questionnaire to measure barriers and facilitators to primary care providers’ involvement

**DOI:** 10.1186/s12875-022-01898-2

**Published:** 2022-12-03

**Authors:** Morgan M. Millar, Teresa Taft, Charlene R. Weir

**Affiliations:** 1 Department of Internal Medicine, University of Utah, 295 Chipeta Way, Salt Lake City, UT USA; 2 Department of Biomedical Informatics, University of Utah, Salt Lake City, UT USA

**Keywords:** Patient recruitment, Clinical trials, Primary care, Surveys, Barriers

## Abstract

**Background:**

Recruitment of sufficient participants for clinical trials remains challenging. Primary care is an important avenue for patient recruitment but is underutilized. We developed and pilot tested a questionnaire to measure relevant barriers and facilitators to primary care providers’ involvement in recruiting patients for clinical trials.

**Methods:**

Prior research informed the development of the questionnaire. The initial instrument was revised using feedback obtained from cognitive interviews. We invited all primary care providers practicing within the University of Utah Health system to complete the revised questionnaire. We used a mixed-mode design to collect paper responses via in-person recruitment and email contacts to collect responses online. Descriptive statistics, exploratory factor analysis, Cronbach’s alpha, and multivariable regression analyses were conducted.

**Results:**

Sixty-seven primary care providers participated in the survey. Exploratory factor analysis suggested retaining five factors, representing the importance of clinical trial recruitment in providers’ professional identity, clinic-level interventions to facilitate referral, patient-related barriers, concerns about patient health management, and knowledge gaps. The five factors exhibited good or high internal consistency reliability. Professional identity and clinic-level intervention factors were significant predictors of providers’ intention to participate in clinical trial recruitment activities.

**Conclusions:**

Results of this exploratory analysis provide preliminary evidence of the internal structure, internal consistency reliability, and predictive validity of the questionnaire to measure factors relevant to primary care providers’ involvement in clinical trial recruitment.

**Supplementary Information:**

The online version contains supplementary material available at 10.1186/s12875-022-01898-2.

## Contributions to the literature

• Tailored implementation strategies are necessary for improving primary care providers’ willingness to refer patients for clinical trials. Yet no existing tool identifies relevant barriers and facilitators to address for a given practice setting.

• This exploratory study presents a promising instrument for measuring barriers and facilitators to providers’ participation in research recruitment activities.

• By providing a systematic method of quantifying relevant barriers to referring patients, this study supports intervention design, thereby assisting in successful adoption and sustainability.

## Background

Failure to recruit sufficient participants is one of the most common reasons for premature termination of clinical trials [1–5]. One analysis found that 57% of terminated trials registered in the clinicaltrials.gov database through 2013 were stopped due to inability to recruit [1] and discontinued trials with poor accrual remain a problem [6]. Between 2000 and 2019, the median sample sizes for completed trials decreased [7]. Clinical trial recruitment remains a priority of the National Institutes of Health’s National Center for Advancing Translational Science [8]. With the additional demands of pragmatic trials, which provide a way for research to be conducted within the point of care [9], the need to support recruitment remains. Furthermore, in the last 2 years the COVID-19 pandemic has highlighted how imperative timely, efficient recruitment is for clinical studies. The pandemic has demonstrated the importance of the ability to quickly develop new treatments in times of crisis [10] and how pandemics or other disruptions may make recruitment for other trials more difficult [11].

Primary care is an alternative avenue for identifying and enrolling research participants. Personal physicians play a critical role in patients’ decision-making and attitudes toward participation in clinical trials [12]. Recruitment of patients for research in primary care settings may be key to faster dissemination and effective translation of research results into practice. However, clinical trial recruitment in the primary care setting remains a challenge [13–16]. Oftentimes providers may agree in principle to assist with recruitment and express favorable attitudes about willingness to recruit [17, 18], but this agreement is a poor predictor of actual behavior [13]. Numerous studies have identified a myriad of physician-reported barriers to participating in research [19–28], ranging from potential patient-related barriers to clinic-level workflow and workload concerns. Our recent qualitative work confirmed many themes identified in the prior literature [29]. We identified six themes among providers’ attitudes toward referral. These included considerations about the perceived benefits and burdens to patients, concern over loss of control over care decisions, the need for proper clinical trial oversight, a lack of time to locate and evaluate trial information, concerns that referral activities may interfere with clinic workflow, and that professional relevance increases motivation to participate in referral.

Identifying the perspectives and beliefs of primary care providers is important in efforts to implement programs for improving primary care physicians’ uptake of clinical trial referral. However, to ensure effectiveness and sustainability, implementation approaches to improve clinical trial recruitment must be tailored to address the specific barriers that are preventing clinicians from engaging in recruitment within a given practice setting [30–32]. Primary care settings are diverse and vary substantially in numerous factors, including available resources for research, attitudes toward research, patient populations, and general workflow. Prior work has focused on qualitative approaches to identifying recruitment obstacles in real time and tailoring implementation strategies to address these barriers [33], but such processes can be time-consuming and costly. Quantitative assessment processes are also needed, both to support a generalizable representation of providers’ mental model of the research recruitment process and to develop tailored implementation strategies.

### Objectives

The purpose of this survey study was to: 1) develop a questionnaire to measure barriers and facilitators to primary care providers referring patients to clinical trials, and 2) conduct initial assessments of the internal structure and predictive validity of the instrument.

## Methods

### Study overview

In this study we created, piloted, and revised a questionnaire. We administered the questionnaire as a paper or online survey of primary care providers. We analyzed results using descriptive statistics, exploratory factor analysis, and a predictive model. See additional file 1, STROBE checklist.

### Item creation

Our approach was guided in part by the Theoretical Domains Framework [31, 34]. The Theoretical Domains Framework of behavior change is a compilation of empirically validated behavioral theories that identifies common constructs and mechanisms of behavior change. It provides a comprehensive summary of domains relevant in influencing health professional behavior. Example domains include knowledge, professional role and identity, beliefs about consequences, and environmental context and resources.

We first identified barriers and facilitators relevant to physicians’ involvement in clinical trial recruitment based on prior research, previous surveys [18, 35, 36], and our own prior qualitative studies [29]. Barriers to provider’s referral of patients to clinical trials in primary care identified in the literature fall into several large categories. First, providers are concerned about how their involvement in research will affect their relationship with patients and whether patients are willing to participate [18, 36–39]. In our prior qualitative study, these concerns were highly prevalent, as providers want to maintain trust with their patients and provide the best care [29]. The second category of barriers concerns the actual time and burden of conducting research in primary care. This factor is frequently found in this literature with primary care providers perceiving increased workload, time constraints, logistics, and clinical workflow factors [28, 35, 36, 39] affecting their ability to participate in research recruitment.

The third category of factors related to providers’ willingness to refer patients is a general lack of comfort regarding research decisions. This in part can be related to a lack of knowledge of research protocols or a lack of awareness of available trials [17, 18, 23, 36]. Further, some providers express unfamiliarity or distrust in researchers or research sites [18, 40]. In our prior interviews with providers, some questioned the quality of the studies and wondered if additional oversight was needed. Finally, the fourth category of barriers is a lack of support in the mechanics of making the referral, which include lack of support staff to assist in the task, the irritation of pop-up alerts, an inability to track patients who have been enrolled, and a lack of feedback about the overall trial and enrollment success [29].

Suggested facilitators or methods to improve primary care provider participation in trial recruitment found in the literature include: 1) organizational strategies that provide logistical support and reduce practitioner workload [13, 37, 38, 41]; 2) simplifying the process for providers [41]; 3) engagement of the researchers with the clinics and providers such as conducting onsite initiation and providing feedback on recruitment outcomes [42]; and 4) the provision of extra research training, protected research time, and individual benefits for providers [13, 23, 29, 38]. We generated an initial set of questionnaire items representing each of the identified barriers and facilitators.

Item construction was guided by best practices in instrument development and survey research. We first compiled a long, diverse set of items with the goal of being as inclusive as possible [43]. We created seven-point construct-specific response scales and minimized the use of the agree/disagree format to better reflect the underlying dimensions being measured [44], to improve item performance [45], and to avoid acquiescence bias and satisficing [46].

### Item revision

Items were further clarified through iterative physician review and cognitive testing. A convenience sample of five primary care physicians were recruited to provide qualitative feedback on the questionnaire content. Three of these interviewees identified as male, and two as female. Three were family medicine providers and two were internal medicine physicians. They were chosen to reflect the variety of experience and expertise in the population of interest. These participants were colleagues of the senior author; two of the interview participants were also included in the survey sample and participated in the subsequent survey. Four of the five had referred patients to clinical trials in the past, but only one had referred more than 25 patients in the past 3 years. All five had experience as a co-investigator in clinical research studies. Each physician participated in a cognitive interview conducted by the first author. They were asked to complete the questionnaire on paper while “thinking aloud” to the interviewer. Interviews lasted approximately 1 hr, and participants were offered a moderate incentive as a mark of appreciation for their time.

In the interviews, we asked participants to explain in their own words the meaning of each item and to explain how they constructed their responses. After completing the questionnaire, we asked providers to provide input on overall clarity and relevance of the items. We used the feedback obtained in the interviews to revise or eliminate problematic items. We conducted five cognitive interviews in total. We concluded conducting cognitive interviews after these five as saturation had been reached; the information obtained from interview participants was very similar.

The interviewees’ feedback led us to cut the length of the survey to increase motivation to complete it by eliminating redundant items and those less relevant to providers’ experiences in referring patients. For example, we cut the number of items measuring the importance of research in the providers’ professional identity in half to reduce redundancy. We eliminated a set of items about comfort in performing general research activities such as conducting literature reviews, designing studies, and interpreting results. We also changed the response scales used for several sets of items to better align with the constructs of interest. For example, we switched from a scale of willingness to refer (how willing or unwilling …) to a scale of likelihood of referral (how likely or unlikely) after finding providers struggling to respond to the willingness scale.

### Questionnaire

The revised questionnaire contained items designed to measure the following domains relevant to providers’ likelihood of participating in clinical trial referral activities: barriers preventing providers from referring patients to clinical trials (nine items), provider concerns about the effects on patient health management (four items), perceived patient-related barriers to clinical trial participation (four items), facilitators that would increase providers’ likelihood of referral (seven items), and importance of research activities to the providers’ professional identity (three items). Another item assessed attitudes about the usefulness of recruiting clinical trial participants via primary care. Each item used a 7-point response scale. See additional file 2, Questionnaire for complete wording of all items.

Other items in the questionnaire included questions asking about providers’ experience with and intentions for referring patients to clinical trials. We examined providers’ experience by asking how many patients they had referred for clinical trials, and if they had previously been an investigator in clinical trial research. We created a set of five items reflecting providers’ reported behavioral intentions in regards to participating in clinical trial referral activities. These items asked how likely the provider was to perform key behaviors associated with involvement in trial recruitment: refer patients to participate in a clinical trial, look for active trials for a patient, conduct preliminary screening of patients to assess trial eligibility, educate patients about participating in trials, and participate in in-service trainings about conducting trials.

We collected information about providers’ professional experience and practice characteristics and basic demographic information. No personally identifying information was requested. The entire survey consisted of up to 45 items with some participants receiving fewer questions due to skip patterns.

### Survey participants and procedures

This project was reviewed and deemed exempt by the University of Utah Institutional Review Board (Review number 00124234). Informed consent was obtained from all participants using a written consent cover letter that appeared at the beginning of the questionnaire. This letter informed participants that participation was voluntary and responses were confidential, and that by completing the survey, they were providing their consent to participate in the study. The survey was administered to primary care physicians and advance practice professionals within the University of Utah Health system during March through December 2020. The advance practice professionals included nurse practitioners (NPs) and physician assistants (PAs)—ordering providers who diagnose and treat illness and provide care, usually under the supervision of a physician. Family practice and internal medicine providers practicing in University of Utah-affiliated community clinics and University of Utah providers with faculty appointments in General Internal Medicine or Family and Preventive Medicine were eligible for the study. To maximize the number of participants, all eligible providers were invited to participate (*n* = 225).

We created both paper and web versions of the questionnaire, using the REDCap survey platform for the online version [47]. These two self-administered modes were chosen to reduce tendencies for socially desirable responses. A subset of the providers (*n* = 12) was invited to participate in the study by the first and senior authors, who visited a regularly scheduled staff meeting for internal medicine clinicians held in March 2020. After a brief introduction, we provided meeting attendees with a paper informed consent letter and a self-administered paper questionnaire. All 12 attendees at this meeting opted to complete the paper questionnaire during the meeting. Due to restrictions put in place at the onset of the COVID-19 pandemic, in-person recruitment ceased after this, and remaining responses were collected online. The remaining providers were emailed to request participation via the online survey. Non-respondents to the initial email received up to two reminder messages.

### Data analysis

Our analysis consisted of three steps, including descriptive analysis, exploratory factor analysis, and a predictive analysis of referral intentions. Cases with missing data on a given variable were excluded when that variable was part of an analysis. We calculated descriptive statistics for each survey item. We conducted an exploratory factor analysis to assess the internal structure of the core questionnaire items. Exploratory factor analysis was chosen over principal components analysis to evaluate the correlation of items with underlying latent constructs and to anticipate unique variance and potential measurement error [48–50]. We present results from a quartimin oblique rotation. We selected an oblique method, rather than an orthogonal, to allow for the anticipated correlation between factors [48, 49, 51]. Other oblique rotation methods produced similar results. For informational purposes, as a comparison, we also ran an a varimax orthogonal rotation and it generated similar results to those presented in this report. Items with a loading of at least |0.50| on a factor were included in each retained factor [52]. We chose |0.50| as a cutoff to provide more stable solutions given our small sample size. Some have suggested factor loadings of |0.60| may be more appropriate for small samples [53]. We opted for |0.50| rather than a stricter cutoff due to the desire to retain more items for future studies of the instrument. As a result, we make note of items loading <|0.60| in our results and the implications are noted in the discussion section below. We retained factors using multiple criteria including eigenvalues greater than one, eigenvalues greater than the point of change in the slope of decreasing eigenvalues as observed in a scree plot, parallel analysis, number of items loading, and theoretical considerations [49, 51, 52].

We calculated internal consistency reliability among the items in each factor using Cronbach’s alpha. We created summary composite scales summing the values of all items loading on each identified factor; items with negative loadings were reverse scored for combination in composite scores. We then evaluated predictive validity by assessing the relationship between these composite variables and providers’ likelihood of referral using linear regression. Analyses were conducted using StataMP, Version 16 (College Station, TX) and SAS 9.4 (Cary, NC).

## Results

### Respondents

Sixty-seven of the 255 (26%) invited providers completed the survey, a response rate consistent with other web surveys of physicians [54]. The remaining invited providers did not respond to multiple requests to participate. The participants who completed the paper survey (*n* = 12) were not significantly different from those responding online in any demographic characteristics or prior trial experience, except for medical specialty. That is, paper participants were all internal medicine specialists.

The responding sample was 58% female and 91% non-Hispanic white (Table 1). Nearly 84% of respondents were physicians, and 15% were advanced practice practitioners. Among physicians, nearly half were board certified in family medicine and 33% were board certified in internal medicine. About 25% of participants had been a principal investigator or co-investigator on a clinical trial in the past, and over half (54%) reported prior experience referring a patient for a clinical trial. Of providers who had referred patients, 72% reported referring five or fewer patients in the last 3 years. These estimates of prior trial referral experience are similar to those reported in prior research [55].

**Table 1:** Characteristics of participants in a survey of primary care providers

	n^**a**^	%
**Ever referred patients to clinical trials**		
No	31	46.3
Yes	36	53.7
**Number of patients referred in last three years**^b^		
5 or fewer	26	72.2
6–25	9	25.0
Over 25	1	2.8
**Clinic participates in trials**		
Yes	33	49.3
No	6	9.0
Not sure	28	41.8
**Experience as a clinical trial investigator (principal investigator or co-investigator)**		
Yes	17	25.4
No	50	74.6
**Years in practice**^c^	13	(5, 20)
**Provider type**		
Physician	56	83.6
Advanced practice provider	10	14.9
Other	1	1.5
**Board certification**^d,e^		
Family medicine	33	49.3
Internal medicine	22	32.8
Pediatrics	8	11.9
Other	2	3.0
**Age**		
Under 40	20	29.9
40–49	25	37.3
50–59	13	19.4
60 or older	9	13.4
**Gender**		
Female	39	58.2
Male	28	41.8

^a)^ Total *n* = 67. There was no missing data for any demographic variables presented

^b)^ Question was only asked of individuals reporting ever referring a patient to a clinical trial

^c)^ Summarized as median (25th, 75th percentile)

^d)^ Question was select all that apply, counts may not sum to total number of participants

^e)^ Only asked of participants reporting a MD or DO degree

### Descriptive statistics

Means and standard deviations and medians with inter-quartile ranges for each core questionnaire item are shown in Table 2. Providers reported higher likelihood of referring a patient to participate in a trial (average of 3.6) than to look for an active trial for their patient (mean = 2.0). The items which were rated the biggest barriers to trial referral were not being aware of what trials exist (mean = 6.2) and no clinic-wide process for identifying appropriate trials (mean = 6.3). Two time-related barriers also appeared to be significant: no time to assess study protocols (mean = 5.7) and no time to discuss research with participants (mean = 5.5).

**Table 2:** Descriptive statistics for barriers and facilitators to referring patients to clinical trials

	Mean (Standard Deviation)
**Likelihood of participating in clinical trial referral activities**	
Refer patients to participate in trial	3.6 (1.7)
Look for active trials for patient	2.0 (1.3)
Conduct preliminary screening for eligibility	2.4 (1.6)
Educate patients about trial participation	2.9 (1.6)
Participate in in-service training about conducting trials	2.6 (1.6)
**Barriers: factors preventing referral of patients to clinical trials**	
No time to assess protocols	5.7 (1.5)
No time to discuss research with patients	5.5 (1.6)
Do not receive recognition for referring patients	3.5 (2.4)
No financial support for participating in referral	3.4 (2.2)
Not aware of what trial opportunities exist	6.2 (1.1)
No clinic-wide process to identify appropriate trials	6.3 (0.9)
Unsure how to evaluate study protocols	4.5 (1.7)
Not knowledgeable about study topic	4.4 (1.7)
Absence of feedback about trial results	3.7 (2.0)
**Barriers: provider concerns about patient health management**	
Study could negatively affect relationship with patients	2.5 (1.7)
Lose control over managing patient’s care	2.4 (1.6)
Uncertainty about health effects of investigational treatments	3.3 (1.7)
Uncertainty about trustworthiness of study sponsor or investigator	2.5 (1.4)
**Barriers: patient-related barriers to referral to clinical trials**	
Patients live too far away from research site	4.1 (1.9)
Study participation is too burdensome for patients	3.9 (1.7)
Patients wary of loss of privacy/confidentiality	3.4 (1.8)
Patients aren’t interested in participating in trials	3.8 (1.5)
**Facilitators: factors that would make clinician more likely to refer patients to clinical trials**	
Introduction to study presented in clinic	4.7 (1.8)
Consistent clinic-level workflow for referring patients	5.2 (1.8)
Electronic health record recruitment alerts	4.3 (2.2)
Documentation of patient enrollment and study outcomes in electronic health record	4.5 (1.8)
Full-time, onsite study coordinator to enroll patients	5.6 (1.7)
Bilingual coordinator to help refer non-English speaking patients	5.0 (1.7)
Dedicated time to participate in research	5.0 (2.0)
**Facilitators: importance of trial referral in professional identity**	
Facilitating clinical trial recruitment is a valuable part of my job	2.4 (1.6)
Referring patients to clinical trials is personally rewarding	2.9 (1.5)
Recommending studies to patients is important to me	3.1 (1.6)

Overall, providers did not express much concern about clinical trial participation affecting their control over patient care, with the average response to these four items ranging from 2.4 to 3.3. Among items questioning patient-related barriers, patients living too far away from research sites was identified as the largest barrier (mean = 4.1).

Responses to items querying whether certain facilitators would increase respondents’ likelihood of referral showed high endorsement with mean values ranging from 4.3 to 5.6. Respondents generally did not see trial recruitment as a valuable part of their job (mean = 2.4).

### Exploratory factor analysis

The results of the exploratory factor analysis and accompanying parallel analysis suggested retaining five factors (Table 3). Eigenvalues for these five factors ranged from 4.31 to 2.48. The first factor included four items representing the relationship of referring patients as part of the importance of trial referral in providers’ professional identity. This factor included all three items that were originally included in the questionnaire “importance of trial referral in professional identity” subscale. Additionally, a time-related barrier, no time to assess protocols, loaded negatively on this factor.

**Table 3:** Factor loadings from exploratory factor analysis

Item	Factor 1	Factor 2	Factor 3	Factor 4	Factor 5
Referring patients to clinical trials is personally rewarding	**0.85**	−0.01	− 0.01	− 0.01	− 0.05
Recommending studies to patients is important to me	**0.78**	0.10	0.08	− 0.18	− 0.07
Facilitating clinical trial recruitment is a valuable part of my job	**0.72**	−0.09	0.11	0.12	−0.17
No time to assess protocols	**−0.60**	0.03	0.20	0.07	−0.05
Electronic health record recruitment alerts	−0.09	**0.86**	−0.04	−0.08	0.03
Health record documentation of patient enrollment and study outcomes	0.01	**0.74**	0.20	−0.03	−0.22
Consistent clinic-level workflow for referring patients	0.30	**0.58**	−0.08	0.05	−0.02
Introduction to study presented in clinic	0.25	**0.53**	−0.13	−0.25	0.00
Do not receive recognition for referring patients	−0.24	**−0.56**	− 0.13	−0.18	− 0.16
No financial support for participating in referral	−0.20	**− 0.59**	−0.04	− 0.23	−0.15
Study participation is too burdensome for patients	0.11	0.08	**0.87**	0.01	0.15
Patients aren’t interested in participating in trials	−0.01	−0.13	**0.83**	−0.01	−0.12
Patients live too far away from research site	−0.09	0.18	**0.81**	−0.01	−0.03
Patients wary of loss of privacy/confidentiality	0.02	−0.10	**0.66**	0.10	0.10
Uncertainty about health effects of investigational treatments	−0.01	−0.01	0.05	**0.84**	−0.13
Uncertainty about trustworthiness of study sponsor or investigator	−0.01	0.16	−0.17	**0.74**	0.09
Study could negatively affect relationship with patients	−0.04	0.00	0.03	**0.74**	0.03
Lose control over managing patient’s care	−0.03	− 0.15	0.09	**0.73**	0.00
Not knowledgeable about study topic	−0.04	−0.03	− 0.08	−0.07	**0.89**
Unsure how to evaluate study protocols	−0.11	−0.05	0.16	0.12	**0.80**
Not aware of what trial opportunities exist	0.01	0.26	0.14	−0.19	**0.54**
Full-time, onsite study coordinator to enroll patients	0.13	0.11	−0.03	0.07	0.12
Dedicated time to participate in research	−0.07	0.10	−0.07	0.13	0.03
Absence of feedback about trial results	−0.20	−0.30	0.29	0.00	0.32
No clinic-wide process to identify appropriate trials	0.29	−0.24	0.06	−0.05	0.38
Bilingual coordinator to help refer non-English speaking patients	0.08	0.19	0.04	0.09	0.22
No time to discuss research with patients	−0.44	−0.23	0.17	0.02	−0.13
Recruiting patients via primary care will improve trial participation	0.44	0.11	0.01	0.02	0.16
**Factor characteristics:**					
Eigenvalue	4.31	4.24	3.23	2.93	2.48
Variance Explained	0.22	0.21	0.16	0.15	0.12
Cronbach’s alpha	0.80	0.85	0.85	0.83	0.77

The second factor represents clinic-level interventions to facilitate referral. Items loading highly on this factor included four clinic workflow or process facilitators that respondents reported could increase their likelihood of referral: incorporating electronic health record recruitment alerts, providing documentation of patient enrollment and study outcomes in the electronic health record, having the study team present an introduction to the study in clinic, and establishment of a consistent clinic-level workflow for referring patients. Two clinic-related barriers, a lack of recognition for referring patients and no financial support for participating in referral, loaded negatively on this factor. Four of these items exhibited factor loadings between >|0.5| and < |0.6|. Using a stricter cutoff of <|0.6|, would have reduced this factor to only two items: incorporating electronic recruitment alerts and health record documentation of patient enrollment and study outcomes.

The third factor, patient-related barriers to referral, consisted of four items all originally conceptualized as factors providers perceive are barriers for patients. These included patients living too far away, studies being too burdensome, patients being wary of loss of privacy, or patients not being interested in clinical trials. The fourth factor was providers’ concern about patient health management. It consisted of the four items that comprised this subscale as we originally conceptualized it, including whether providers expressed concern about a study negatively affecting their relationship with patients, losing control over managing patients’ care, uncertainty about health effects of investigational treatments on patients, and uncertainty about the trustworthiness of the study. Factor five consisted of three items representing barriers to referral related to knowledge gaps. These included not being aware of what trials exist, being unsure about how to evaluate study protocols, and not being knowledgeable about the study topic. The factor loading for the item ‘lack of awareness of trial opportunities’ was less than < 0.6.

Preliminary model fit statistics included a root mean squared error of approximation of 0.10, a comparative fit index of 0.83, a Tucker-Lewis index of 0.80, and standardized root mean squared residual of 0.12. A matrix displaying correlations between the five identified factors is displayed in Table 4. The factor ‘importance of trial referral to providers’ professional identity’ was significantly correlated with the factor representing ‘clinic-level interventions to improve referral’ (0.48, *p* < 0.001). The ‘patient-related barriers’ factor was slightly correlated with ‘providers’ concerns about patient management’ (0.21) and ‘providers’ knowledge gaps about trial referral’ (0.22), but not significantly.

**Table 4:** Correlation between factors identified in exploratory factor analysis

Factor	Importance of referral	Clinic-level interventions	Patient barriers	Patient management	Knowledge gaps
Importance of referral	1.0				
Clinic-level interventions	0.48	1.0			
Patient barriers	−0.07	0.04	1.0		
Patient management	− 0.14	0.01	0.21	1.0	
Knowledge gaps	−0.17	0.08	0.22	0.07	1.0

Several items did not load highly on any of the factors, including provision of full-time study coordinators, dedicated time to participate in research, bilingual coordinators to help with recruitment, and an item measuring attitudes about the usefulness of primary care recruitment. These items were eliminated from the questionnaire. Other items did not load highly enough on a single factor but instead exhibited moderate loadings on multiple factors These items included: absence of feedback about trial results, no clinic-wide process to identify trials, and not time to discuss research with patients. These items were also eliminated.

### Internal consistency reliability

We explored initial internal consistency reliability of the proposed factors. Internal consistency for the first four factors was high; each had a Cronbach’s alpha over 0.80 (Table 3), indicating high internal consistency reliability. The fifth factor’s internal consistency was acceptable, just missing the 0.80 threshold with an alpha of 0.77.

### Predicting intentions

We explored the predictive validity of the instrument by evaluating the relationship between providers’ intentions to participate in referral and the five summary variables representing the proposed factors. Behavioral intentions were captured using the five likelihood of referral activity items. These five items had very high internal consistency reliability (alpha = 0.89) and maintained a single factor solution with high factor loadings when included in an exploratory factor analysis. Using bivariate correlations, we observed that likelihood of referral was significantly correlated with the first two factors, importance of referral in professional identity (0.65, *p* < 0.001) and clinic-level interventions (0.62, *p* < 0.001). These two variables were then both included in a multivariable linear regression predicting referral intentions, Table 5. Both variables were significant predictors of providers’ likelihood of participating in patient referral activities in the multivariable model.

**Table 5:** Relationship between identified factors and providers’ likelihood of participating in clinical trial referral activities^a^

	Coefficient	Robust Standard Error	*P*	95% Confidence Interval	
Variable:					
Importance of referral	0.61	0.16	**< 0.001**	0.28	0.94
Clinic-level interventions	0.28	0.07	**< 0.001**	0.15	0.42
Constant	−0.99	1.48	0.507	−3.94	1.97

^a^ Assessments indicated no violation of assumptions of linear regression. Visual patterns in scatterplots confirmed linear relationships, Shapiro-Wilk *p* = 0.99 for multivariate normality, variance inflation factors < 5 indicate little multicollinearity, the Durbin-Watson coefficient 2.06 indicates no autocorrelation, and residual plots indicate homoscedasticity

## Conclusions

This paper presents the results of an exploratory factor analysis of a newly created survey instrument to measure barriers and facilitators to providers participating in recruitment activities for clinical trials. Our results provide preliminary evidence of the utility of this questionnaire designed to measure constructs identified in prior research. Items loaded onto five factors in a logical way that is largely consistent with the literature and our original conception of the questionnaire, and those that did not have been eliminated from the revised questionnaire. Internal consistency for these subscales was high. Furthermore, the likelihood of participating in trial referral activities variable we created demonstrated high internal consistency reliability. Our results provide promising preliminary evidence for the internal structure of this questionnaire. However, these results are preliminary. More research is now needed to further evaluate and refine the instrument. Our exploratory findings should be tested in larger and more diverse samples. Additionally, additional validity assessments and confirmatory factor analysis are needed.

In our assessment of the questionnaire, some of the factor analysis results were somewhat unexpected. We found that a time-related barrier to referral (no time to assess study protocols) loaded negatively on the first factor alongside items representing the importance of referral as part of the clinician’s professional identity. This result is understandable as individuals are only likely to try to make time for participating in research recruitment activities if they find these activities important and worthwhile. Two items we had framed as barriers to referral, the lack of recognition for referring patients and lack of financial support for participating in clinical trial referral, also loaded negatively on the second factor, clinic-level interventions to improve participation in trial referral. These are two factors that, if framed positively, would be clinic-based approaches to supporting clinicians in participating in research recruitment. Policies such as providing clinicians with recognition and financial support, such as research relative value units, for referral participation could be facilitators that would improve their participation. Several barrier items also did not load highly on any factors and could be eliminated from the revised questionnaire.

In our model predicting referral intentions, providers’ professional identity and clinic-level interventions to improve referral were associated with higher willingness to participate in research referral. The association between identity and behavioral intentions is consistent with theoretical models of behavior change [31]. Clinic-level interventions may be key to engaging primary care clinicians in research recruitment and improving trial enrollment. Numerous studies have identified a myriad of physician-reported barriers and facilitators to participating in research [19–28]. In the face of such barriers, programs to improve provider involvement in trial recruitment must be designed to address the local, clinic level barriers. In the implementation planning process, health systems must find ways to identify relevant barriers to target to better engage clinicians in the trial enrollment process. Research into the most effective solutions to overcome these barriers is ongoing and include informatics interventions, professional development, and clinic workflow remodeling.

Several advances have been made in designing informatics solutions to support clinical trial recruitment, including tools for identifying cohorts of eligible patients. For example, among other goals, the Accrual to Clinical Trials Network—a consortium of multiple National Clinical and Translational Science Award sites aimed at increasing patient accrual to multisite trials [8]—is establishing a digital infrastructure to allow for multisite identification of eligible patients for trials. Similarly, multiple research sites are now using tools such as the Electronic Medical Record Search Engine to support cohort discovery [56]. Machine learning algorithms are increasing the efficiency of eligibility screening and reducing the number of patients that necessitate manual review of charts to establish eligibility [57].

Other informatics solutions include alerts to providers of patient eligibility. Real-time eligibility alerts are showing promise for identifying patients while in-clinic [58], and evidence suggests that patient screening alerts improve patient screening efficiency and lead to higher enrollment [59]. The Accrual to Clinical Trials Network also aims to assist providers in identifying relevant trials for their patients [8]. However, there is wide variation in how informatics solutions are implemented, as workflow and regulatory processes vary and approaches to implementation differ [60]. More work is still needed to develop best practices. Furthermore, sustainability of interventions such as targeted alerts remains an issue, with evidence suggesting that provider responsiveness to clinical trial alerts declines over prolonged exposure [61]. Targeting relevant barriers will help ensure success and sustainability of intervention.

There have been few high-quality trials to test interventions to improve clinicians’ recruitment activities. One systematic review found some evidence in favor of reducing clinical workload, improving training, and provision of protected research time, but also noted that more high quality evaluations of interventions are needed [62]. Recruiter training sessions have been suggested to overcome knowledge barriers or discomfort clinicians may have about communicating about randomized trials to patients. However, there is little evidence of effective interventions aimed at training clinicians in clinical trial recruitment [63]. One proposed solution to incentivize clinicians is the allocation of relative value units for research-related activities, but we still have not adequately addressed how to incentivize providers to participate in research recruitment [64].

Pragmatic trials that randomize at the point of care are a solution to many challenges to conducting clinical trials. They may address the recruitment challenges because randomization is automatic and the clinical experience for both patients and providers is essentially similar to that for patients who have not been randomized to a treatment. However, providers must be willing to participate. Our prior research suggests primary care providers perceive a variety of barriers to point-of-care research and are skeptical about the concept of equipoise in studies comparing two treatment approaches [39]. Pragmatic trials may necessitate providers going against their own established standards of care or those that are embedded into the culture of their clinical practice.

A limitation of this study is its small sample size, which may have resulted in imprecise estimates. We initially planned to conduct more in-person recruitment, which we believe would have resulted in higher response, but the onset of COVID-19 prevented us from continuing with this approach. The sample size to number of items ratio is also a significant limitation [53], so results must be replicated with larger samples [65]. With this in mind, like other small-sample studies, we employed a higher factor loading cutoff (>|0.5|) which can allow for stable factor solutions from much smaller samples [66]. We also considered an even stricter cut-off of >|0.6|, and have noted how this would have eliminated additional items. We believe these items loading between 0.5 and 0.59 warrant further investigation in future assessments of the questionnaire, and so opted to retain them for purposes of future evaluation. Also, several items had moderate loadings on several factors and thus were not discriminating or good items. Poor question wording could have introduced imprecise measurement, so we opted to eliminate these items.

Another limitation is that this exploratory study was conducted within a single healthcare system and only 26% of the sample participated. Thus the results may not be generalizable to the full sample, and the providers in our study may not be representative of primary care practitioners more broadly in their experiences with clinical trials or attitudes toward referring patients. Our study was conducted within an academic medical center, whereas a majority of primary care physicians in the United States work in private, physician-owned practices [67]. Our sample was also over-representative of MD/DO providers compared to advanced practice providers (MD/DOs were 84% of our sample, 68% nationally) and female practitioners were overrepresented (58% compared to 45% nationally) [67]. One-quarter of participants had prior experience as a co-investigator or principal investigator in clinical research, and just over half reported prior experience referring patients to trials. Yet of those who had referred patients, most had not referred more than 25 patients in the last 3 years. If our sample overrepresents those with significant trial experience, this could have resulted in underestimates of certain barriers or limited our ability to ascertain the relevance and predictive validity of specific barriers. For example, only two of our five identified factors were predictive of providers’ reported likelihood of participating in referral activities. It is plausible that in other clinical contexts additional factors would be significantly affecting provider behavior. Thus, these results are not conclusive and continued evaluation of this instrument is necessary to assess the generalizability of our findings, the fit of our model, and further assess the validity of the questionnaire using larger samples of clinicians from a variety of healthcare systems.

Nevertheless, the exploratory analyses of the instrument provide initial evidence of the internal structure and suggests high internal consistency reliability. We believe the results warrant further evaluation of its validity. Our goal is for this instrument to be a useful tool in implementation planning in healthcare settings. Furthermore, we also foresee the use of the questionnaire to assess whether attitudes and barriers change over time. The domains contained in the instrument will remain relevant, but what issues are the most pressing are likely to change over time within a given health system. Repeated administration of the instrument, over time, may also assist in intervention effectiveness and sustainability. Continued learning, problem solving, and adaptation [68] over time can ensure that interventions designed to improve trial recruitment activities continue to be relevant to the context.

Improving trial enrollment is critical. Primary care referral can help address difficulties in recruiting patients for trials and has the potential to allow for quicker dissemination of science into care. Researchers must now concentrate on designing and evaluating interventions that effectively address the barriers clinicians face in participating in the recruitment process.

## Supplementary Information

**Additional file 1.** STROBE checklist

**Additional file 2.** Questionnaire

## Data Availability

The dataset generated for this study are not publicly available due to confidentiality provisions provided to participants upon enrollment in the study. Data are available from the authors upon reasonable request with approval by the University of Utah Institutional Review Board.
