# Supplementary material for: Clinical trial recruitment in primary care: exploratory factor analysis of a questionnaire to measure barriers and facilitators to primary care providers’ involvement
Source: BMC Prim Care. 2022 Dec 3;23:311. doi: 10.1186/s12875-022-01898-2 (PMC9719201; doi:10.1186/s12875-022-01898-2)
Supplement: Supplementary file 2 — Additional file 2. Questionnaire [file 12875_2022_1898_MOESM2_ESM.docx]

**Clinical trial recruitment in primary care: care: factor analysis of a questionnaire to measure barriers and facilitators to primary care providers’ involvement**

**Study Questionnaire**

**Note: Items not loading on retained factors marked with an asterisk (*)**

**Recruiting for Clinical Trials in Primary Care**

Thank you for your help with this survey. The purpose of this study is to understand barriers and facilitators associated with referring patients for participation in clinical trials via primary care. Your responses will help us identify how clinics and health systems can better support clinicians in clinical trial referrals.

1. **Have you ever referred a patient for participation in a clinical trial?**

- Yes
- No 🡪 **Skip to Question 2**

**1a. Approximately how many patients have you referred to a clinical trial *in the last 3 years*?**

- 5 or fewer
- 6-25
- 26 or more

1. **Does your primary care clinic participate in clinical trials?**

- Yes
- No
- Not sure

1. **Have you ever been a co-investigator (Co-I) or a principal investigator (PI) for a clinical trial? *Select all that apply:***

- Co-investigator (Co-I)
- Principal Investigator (PI)
- Neither

1. **Given the *demands and resources of your current clinical practice*, how likely or unlikely are you to do each of the following? *Circle the number that best corresponds to your response*.**

|  | Very unlikely |  |  |  |  |  | Very likely |
| --- | --- | --- | --- | --- | --- | --- | --- |
| 1. Refer your patients to participate in clinical trials | 1 | 2 | 3 | 4 | 5 | 6 | 7 |
| 1. Look for active trials for your patients | 1 | 2 | 3 | 4 | 5 | 6 | 7 |
| 1. Conduct preliminary screening to assess if your patients are eligible for a clinical trial | 1 | 2 | 3 | 4 | 5 | 6 | 7 |
| 1. Educate patients about participating in clinical trials | 1 | 2 | 3 | 4 | 5 | 6 | 7 |
| 1. Participate in in-service training about conducting clinical trials | 1 | 2 | 3 | 4 | 5 | 6 | 7 |

1. **To what extent does each of the following issues *prevent* you from referring more patients for clinical trials? *If an issue is not applicable to you, please mark “not at all”***

|  | Not at all |  |  |  |  |  | Very much |
| --- | --- | --- | --- | --- | --- | --- | --- |
| 1. No time to assess study protocols | 1 | 2 | 3 | 4 | 5 | 6 | 7 |
| 1. No time to discuss research participation with patients* | 1 | 2 | 3 | 4 | 5 | 6 | 7 |
| 1. No recognition for referring patients | 1 | 2 | 3 | 4 | 5 | 6 | 7 |
| 1. No financial support for participating in referral | 1 | 2 | 3 | 4 | 5 | 6 | 7 |
| 1. Not aware of what trial opportunities exist | 1 | 2 | 3 | 4 | 5 | 6 | 7 |
| 1. No clinic-wide process to identify appropriate trials* | 1 | 2 | 3 | 4 | 5 | 6 | 7 |
| 1. Unsure how to evaluate study protocols | 1 | 2 | 3 | 4 | 5 | 6 | 7 |
| 1. Not knowledgeable about the study topic | 1 | 2 | 3 | 4 | 5 | 6 | 7 |
| 1. Absence of feedback about trial results* | 1 | 2 | 3 | 4 | 5 | 6 | 7 |

1. **To what extent is each of the following a *concern* you would have about referring your patients to participate in clinical trials?**

|  | Not at all a concern |  |  |  |  |  | A signif-icant concern |
| --- | --- | --- | --- | --- | --- | --- | --- |
| 1. The study could negatively affect your relationship with patients | 1 | 2 | 3 | 4 | 5 | 6 | 7 |
| 1. You might lose control over managing your patients’ care | 1 | 2 | 3 | 4 | 5 | 6 | 7 |
| 1. Uncertainty about health effects of investigational treatments | 1 | 2 | 3 | 4 | 5 | 6 | 7 |
| 1. Uncertainty about the trustworthiness of the study sponsor or investigator | 1 | 2 | 3 | 4 | 5 | 6 | 7 |

1. **To what extent would each of the following *patient-related factors* be a barrier to you referring them to participate in clinical trials?**

|  | Not at all a barrier |  |  |  |  |  | A signif-icant barrier |
| --- | --- | --- | --- | --- | --- | --- | --- |
| 1. Patients often live too far away from research sites | 1 | 2 | 3 | 4 | 5 | 6 | 7 |
| 1. Study participation is too burdensome for patients | 1 | 2 | 3 | 4 | 5 | 6 | 7 |
| 1. Patients are wary of loss of privacy or confidentiality | 1 | 2 | 3 | 4 | 5 | 6 | 7 |
| 1. Patients aren’t interested in participating in trials | 1 | 2 | 3 | 4 | 5 | 6 | 7 |

1. **To what extent would each of the following make you *more likely* to refer patients to a clinical trial?**

|  | Would not increase my likelihood at all |  |  |  |  |  | Would make me much more likely |
| --- | --- | --- | --- | --- | --- | --- | --- |
| 1. An introduction of the study presented to your clinic | 1 | 2 | 3 | 4 | 5 | 6 | 7 |
| 1. A consistent clinic-level workflow for referring patients | 1 | 2 | 3 | 4 | 5 | 6 | 7 |
| 1. EHR recruitment alerts | 1 | 2 | 3 | 4 | 5 | 6 | 7 |
| 1. EHR documentation of patient enrollment and study outcomes | 1 | 2 | 3 | 4 | 5 | 6 | 7 |
| 1. A full-time onsite study coordinator to enroll patients* | 1 | 2 | 3 | 4 | 5 | 6 | 7 |
| 1. A bilingual coordinator to help refer non-English speaking patients* | 1 | 2 | 3 | 4 | 5 | 6 | 7 |
| 1. Dedicated time to participate in research* | 1 | 2 | 3 | 4 | 5 | 6 | 7 |

1. **To what extent do you agree or disagree with the following statement?**

|  | Strongly disagree |  |  |  |  |  | Strongly agree |
| --- | --- | --- | --- | --- | --- | --- | --- |
| 1. Recruiting patients via primary care clinics will significantly increase clinical trial participation | 1 | 2 | 3 | 4 | 5 | 6 | 7 |

1. **To what extent does each of the following statements describe you in your current professional situation:**

|  | Does not describe me at all |  |  |  |  |  | Describes me very much |
| --- | --- | --- | --- | --- | --- | --- | --- |
| 1. Facilitating clinical trial recruitment is a valuable part of my job | 1 | 2 | 3 | 4 | 5 | 6 | 7 |
| 1. Referring patients for clinical trials is personally rewarding for me | 1 | 2 | 3 | 4 | 5 | 6 | 7 |
| 1. Recommending research studies to my patients is important to me | 1 | 2 | 3 | 4 | 5 | 6 | 7 |

*Finally, we would like to learn a bit more about you and your practice setting.*

1. **How many years have you practiced in primary care?** ______Years
2. **Approximately how many patients do you have?** _______Number of patients
3. **On average, how many minutes do you spend in each primary care patient visit?** ______ Minutes
4. **What type of certification/degree do you have?**

- MD or DO
- Advanced Practice Clinician (e.g., PA-C, APRN) 🡪 **Skip to Question 15**
- Other, please specify: _______________________________

**14a. Are you board certified or board eligible in any of the following specialties? *Select all that***

***apply.***

- Family medicine
- Internal medicine
- Geriatrics
- Pediatrics
- OB/Gyn
- Other, please specify: ___________________________

1. **What is your age?**

- Under 30
- 30-39
- 40-49
- 50-59
- 60-69
- 70-79
- 80+

1. **What is your gender?**
   - Male
   - Female
   - Non-binary
2. **Which of the following describes your race or ethnicity? *You can select as many as apply.***
   - Asian
   - Black or African American
   - Hispanic or Latino/a
   - Native American, American Indian, or Alaska Native
   - Pacific Islander or Native Hawaiian
   - White
   - Other, please specify: __________________________________

**Thank you so much for your responses! If you have any other thoughts you’d like to share with us about referring patients for clinical trials, please share them here:**
